# Inverse Correlation of Population Similarity and Introduction Date for Invasive Ascidians

**DOI:** 10.1371/journal.pone.0002552

**Published:** 2008-06-25

**Authors:** Nathan Silva, William C. Smith

**Affiliations:** 1 Interdepartmental Graduate Program in Marine Science, University of California Santa Barbara, Santa Barbara, California, United States of America; 2 Department of Molecular, Cellular and Developmental Biology, University of California Santa Barbara, Santa Barbara, California, United States of America; University of California, Berkeley, United States of America

## Abstract

The genomes of many marine invertebrates, including the purple sea urchin and the solitary ascidians *Ciona intestinalis* and *Ciona savignyi*, show exceptionally high levels of heterozygosity, implying that these populations are highly polymorphic. Analysis of the *C. savignyi* genome found little evidence to support an elevated mutation rate, but rather points to a large population size contributing to the polymorphism level. In the present study, the relative genetic polymorphism levels in sampled populations of ten different ascidian species were determined using a similarity index generated by AFLP analysis. The goal was to determine the range of polymorphism within the populations of different species, and to uncover factors that may contribute to the high level of polymorphism. We observe that, surprisingly, the levels of polymorphism within these species show a negative correlation with the reported age of invasive populations, and that closely related species show substantially different levels of genetic polymorphism. These findings show exceptions to the assumptions that invasive species start with a low level of genetic polymorphism that increases over time and that closely related species have similar levels of genetic polymorphism.

## Introduction

Ascidians are invertebrate chordates exclusive to the marine environment. Many ascidian species have been classified as invasive and/or bio-fouling, and will rapidly colonize empty substrates in harbor environments, and are especially problematic for facilities culturing marine organisms [1], [2]. Ascidians can also reduce the amount of biodiversity in ecosystems into which they are introduced [3]. Adult ascidians are sessile and accomplish the majority of their dispersal as larvae. Two general strategies of larval production are observed in ascidians: broadcast spawning and brooding. Ascidians that broadcast spawn send their gametes into the water column where fertilization and subsequent development occur. Broadcast spawning is generally a characteristic of solitary ascidian species. Conversely, colonial ascidian species (i.e., species that can reproduce asexually by budding, as well as sexually) typically brood their embryos until hatching. Broadcast spawning results in non-feeding embryos and larvae that have a residence time measured in days in the planktonic community [4]. Brooding ascidians on the other hand, undergo internal fertilization and development so that a fully-formed non-feeding swimming tadpole larva is released from the adult [5]. These brooded larvae have a planktonic residence time measured in hours rather than days and thus have shorter potential dispersal distances compared to the broadcast spawned larva [6].

Due to their bio-fouling behavior, adult ascidians, which frequently settle on boats, are likely to become transient members of multiple marine communities. It is possible that even a single ship-borne ascidian could start a new population as ascidians are hermaphrodites and many are able to self-fertilize. Non-self sperm is typically much more effective than self sperm during fertilization, a mechanism which supports out-crossing when multiple ascidians of the same species are in a single location, but this potential for self fertilization may help establish ascidian colonies in new locales [7].

Many species of ascidians are known to be invasive to the Southern California Bight (SCB) [8], [9]. As early as 1915, non-native ascidian species were identified and recorded [10]. Current surveys show that 14 species of invasive ascidians have established populations along the Southern Californian Bight [8], [9]. These introduction events are hypothesized to occur by anthropogenic means, either through fouled ships or ballast water introduction. The most recently recorded introduction into the SCB was in 1997 [9]. Once introduced, these invasive species can persist for many years [8], [9]. With predicted increases of sea-surface temperature, more tropical ascidian species will likely also be introduced and established in the harbors of the SCB as well as other harbors throughout the world [11].

Two common invasive ascidians are the related species *Ciona savignyi* and *Ciona intestinalis*, which are becoming important model systems for experimental biology. Recent genomic sequencing of these two species revealed extremely high levels of genetic polymorphism in samples collected from California, where they are not thought to be native [12], [13]. *C. savignyi* is reported to display a 4.6% difference between haploid genomes at the nucleotide level [13] which is comparable to the amount of genetic difference between humans and old world monkeys [14]. The nucleotide difference within the haploid genomes of *C. intestinalis* is closer to 1.2% which is still quite polymorphic when compared to other sequenced genomes [12]. The origin of the high polymorphism in these two ascidian species has been investigated, and much of the polymorphism appears to be attributable to the large population sizes [15]. An even higher level of polymorphism has been found in the purple sea urchin *Strongylocentrotus purpuratus* [16], suggesting that such high levels of polymorphism may be more common than previously thought. However, outside of the two *Ciona* species, little or no sequence information for ascidians is available.

The goal of this project was to examine the relative degrees of genetic polymorphism in ten different species of ascidians found at two southern California locations, the Santa Barbara Yacht Harbor and the Ventura Harbor. All the species chosen are abundantly represented at these two locations [8], [9]. The ascidian species selected encompass a range of traits, evolutionary histories, and population histories. This study included highly divergent clades of ascidians (*Stolidobranchia*, *Phlebobranchia*, and *Aplousobranchia*) [17], species with differing reproductive strategies (broadcast spawning versus brooding), solitary and colonial species, and finally species that are thought to be endemic to southern California versus introduced species [8].

For determining relative polymorphism levels from these ten species we used an AFLP-based strategy, rather than directly generating nucleotide sequence data. Sequence data would by necessity be limited to only a few genetic loci for each species. Furthermore, sequence data would be restricted to those loci for which degenerate PCR primers could be found to work in all ten species, which could potentially bias the results to more highly conserved loci. AFLP on the other hand gives a mostly unbiased “fingerprint” of amplified restriction fragments from multiple loci throughout the genome [18]. Accurately assessing the amount of genetic polymorphism within many species of ascidians will give an indication of the extent and variability of this high polymorphism phenomenon, and may give an indication of its cause and significance.

## Materials and Methods

### Sample Collection

**Figure 1:**
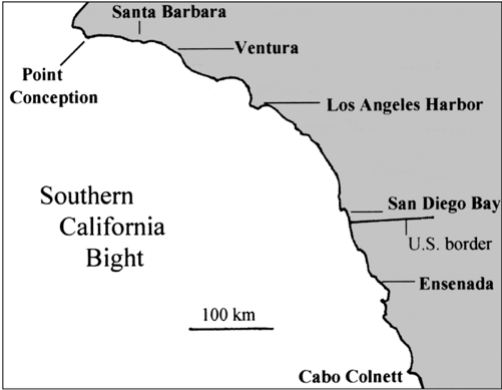
A map of the Southern California Bight modified from [9]. The relative locations of the Santa Barbara Yacht Harbor and Ventura Harbor are shown.

Fifty individuals from ten species of ascidians (Table 1) were collected in the Santa Barbara Yacht Harbor (SBYH) located at 34°25′N; 119°42′W. SBYH is located 78 km south of Point Conception, California and 39 km northwest of Ventura Harbor (VH) [8]. 50 individuals of both *C. intestinalis* and *C. savignyi* were also collected from VH, which is located at 34°17′N; 119°18′W (Fig. 1)[8]. All animals of a species were collected within three months of each other and the entire collection process occurred between January 2006 and September 2007. For each species no more than three individuals were collected within 30 cm of each other and no species had all of their samples collected in a single day. Colonial ascidians were collected no closer than 10 cm from the next colony of the same species to avoid collecting clones.

**Table 1:** Ascidian species analyzed in this study.

Species	Taxonomy	Body Plan	Fertilization Method	Date First Recorded	Sequenced Genome
*Aplidium californicum*	Aplousobranch	Colonial	Brooding	Native	No
*Ascidia ceratodes*	Phlebobranch	Solitary	Broadcast-spawning	Native	No
*Ascidia zara*	Phlebobranch	Solitary	Broadcast-spawning	1984	No
*Botrylloides diegensis*	Stolidobranch	Colonial	Brooding	Native	No
*Botrylloides violaceus*	Stolidobranch	Colonial	Brooding	1945	No
*Botryllus schlosseri*	Stolidobranch	Colonial	Brooding	1965	No
*Ciona intestinalis*	Aplousobranch	Solitary	Broadcast-spawning	1915	Yes
*Ciona savignyi*	Aplousobranch	Solitary	Broadcast-spawning	1985	Yes
*Styela clava*	Stolidobranch	Solitary	Broadcast-spawning	1933	No
*Styela plicata*	Stolidobranch	Solitary	Broadcast-spawning	1915	No

Date first recorded is based on the surveys and publication of the Lamberts [8], [9]. The taxonomic classification is according to Turon and López-Legentil [17].

### DNA Extraction

Genomic DNA was isolated from siphon muscle or sperm of solitary ascidians, and dissected body wall tissue of colonial ascidians, with special care taken to avoid the gastro-intestinal tract and developing larvae. Tissue was first incubated overnight at 55°C with agitation in a digestion buffer of 100 mM NaCl, 20 mM Tris pH 7.8, 10 mM EDTA, 1% SDS, and proteinase-K at 250 µg/ml. Samples were then homogenized with a microtube pestle to break up any undigested tissue and incubated further. After two rounds of 50∶50 phenol/chloroform extraction and one round of chloroform extraction, samples were ethanol precipitated. The resulting pellets were resuspended in TE buffer overnight at 4°C, followed by an RNase A digestion. Finally, the DNA was purified further using glass milk (Q-BIOgene, Gene Clean III Kit). The sample of genomic DNA was then analyzed by 1% agarose-gel electrophoresis to determine its quality and quantity. Samples with high quality/high molecular weight genomic DNA were quantified on a spectrophotometer and diluted or concentrated to ∼100 ng/µl.

### AFLP

Genomic DNA samples were restriction enzyme-digested and ligated with adaptors using the Invitrogen AFLP Analysis System II kit as described in the manual, with the exception that no denaturation was performed prior to ligation, as described by Bonin *et al.* [19]. Adaptor and preselective PCR primer sequences were as described in Vos *et al.* [18]. For the preselective amplification twenty cycles of 94°C for 20 sec, 56°C for 30 sec, 72°C for 2 min were performed, followed by incubations at 72°C for 2 min and 60°C for 30 min. The pool of fragments generated were then diluted and used as the template for the second round of selective amplification. Two separate selective amplification were performed using either primer sets **E-AA**: 5′-GACTGCGTACCAATTCAA-3′ and **M-CAG**: 5′-GATGAGTCCTGAGTAACAG-3′, or **E-TG**: 5′-GACTGCGTACCAATTCTG-3′ and **M-CTC**: 5′-GATGAGTCCTGAGTAACTC-3′. The E-AA and E-TG primers were labeled with Well-Red D4 dye (Promega). The selective amplification program consisted of an initial denaturation step of 94°C for 2 min, 10 cycles of 94°C for 20 sec, an annealing temperature for 30 sec, starting at 66°C and lowering by one degree per cycle to a final annealing temperature of 56°C, and an extension of 2 min at 72°C. After this initial 10 cycles of the “touch down” phase of the selective amplification, 25 cycles of 94°C for 30 sec, 56°C for 30 sec and 72°C for 3 min were performed, and were followed by a final incubation for 30 min at 60°C.

### Fragment analysis

The products of the selective amplification were analyzed on a CEQ 8000 fluorescent capillary sequencer (Beckman Coulter). One µl of the sample was added to 38.5 µl of Sample Loading Solution and 0.5 µl of Well-Red D1 dye-labeled GenomeLab DNA Size Standard Kit – 400 (Beckman Coulter). The samples were run according to the default Frag-3 run method, which consists of a 30 sec injection of sample at 2.0 kV and a 35 min run at 6.0 kV. The resulting data were then exported for use with Genographer software for further analysis [20].

### Data Analysis

The fluorescent trace data were imported into Genographer as individual waveforms, and converted into a synthetic gel for visualization of the results. The program aligned the various samples by use of the labeled size-standards that were run with each sample. Once imported, the samples were normalized and the default intensity was increased five-fold. Bins of ∼1 bp in length were manually selected across all samples of a population wherever visual bands were present within the 60 to 350 bp size range. This procedure was performed in an identical manner with all populations analyzed. Each bin was treated as a separate genetic marker. The waveforms at each marker were visualized using the “thumbnail” option within Genographer. These waveforms were then visually scored for peaks within the bin and recorded as either present or absent in a binary fashion. The qualifications for calling a peak were that the peak of fluorescent intensity must be within the selected bin and that the relative intensity must be above the background. Each bin was recorded as a separate genetic marker, and will be referred to as “marker”. The presence of a DNA fragment in the bin for each marker (i.e. presence allele) was recorded and will be referred to as “band”.

Data from the similarity matrix were resampled as five groups of five and ten randomly chosen individuals. The resulting distributions of marker frequency from the small randomly chosen groups were plotted and compared to the results from the analysis performed on the complete set of 50 individuals.

### Similarity Index

The similarity index consisted of rows representing markers and columns representing individuals. The presence of bands was recorded in a binary manner, with “1” signifying presence and “0” signifying absence. In this manner, each marker would have a value representing the number of individuals in the sampled population which shared the marker (the sum of the row) and each individual would have a value representing the total number of markers scored for that individual (the sum of the column).

### Population Structure Analysis

The AFLP-SURV software was used to determine the amount of genetic distance between the SBYH and VH populations of both *C. intestinalis* and *C. savignyi* [21]. The 50 individuals from both *C. intestinalis* populations (n = 100 total) were analyzed simultaneously using the Genographer method mentioned above so that all loci scored were scored in both populations, and so that the identity of the markers used were consistent between the two populations. The same combined analysis was done for the *C. savignyi* populations (n = 100). The analysis performed on these data was the Bayesian method with non-uniform prior distribution. The F_ST_ values generated by this analysis were recorded as a measure of genetic distance and population structure between the two populations of each species.

## Results

### Validation of AFLP technique

In order to validate the use of AFLP for this study we first determined the experimental reproducibility of the markers generated from multiple DNA samples prepared from a single *C. savignyi*. From the one *C. savignyi* individual processed and assayed as six separate samples, 52 AFLP markers were identified (data not shown). Although the intensity of the markers was somewhat variable between samples, 49 out of 52 markers were monomorphic (i.e., present in all samples), while the remaining three markers were found in five of the six samples. We thus concluded that our AFLP methodology gave highly robust markers.

### Ascidian species show varying degrees of polymorphism

**Figure 2:**
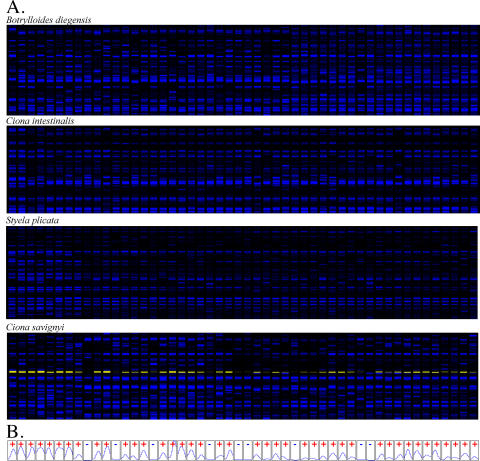
Representative data as displayed by the Genographer program. A. Synthetic gels generated by AFLP for 4 different species from SBYH (*C. savignyi*, *C. intestinalis*, *Styela plicata*, and *Botrylloides diegensis*). A window with fragments in the range of 75 bp–150 bp is shown. B. Representative scoring of bands in a typical marker in *Ciona savignyi* (shown in yellow).

Genomic DNA was isolated from 50 individuals collected at SBYH for the ten species listed in Table 1. In addition, genomic DNA was collected from 50 *C. savignyi* and *C. intestinalis* from VH, located approximately 39 km south-east of Santa Barbara (Fig. 1). Samples from all 50 individuals of each species were processed and analyzed as described in Materials and Methods. The data was recorded as a similarity index in which the presences of AFLP bands of specific sizes (referred to here a “markers”) were scored for each sample. Figure 2A and B give examples of the data and scoring of a typical band. It was observed that the presence or absence of a particular marker was more variable than the number of markers scored per individual, as would be expected for individuals within a species having the same genome sizes and compositions, but differing alleles.

The results for the analysis of all ten species are summarized in Table 2A. The number of scoreable markers summed for the 50 individuals is given in the second column. The value of the total marker number between species should vary depending on the genetic polymorphism within the population sampled, as well as the size and composition of the genomes (i.e., the frequency of the recognition site for the restriction enzymes) and ranged from 389 for *Botrylloides diegensis* to 175 for *Ciona intestinalis*. The average number of bands per individual (i.e., average number of presence alleles for all possible markers) varied from 103±16 for *Ascidia zara* to 228±15 for *Botryllus schlosseri*. The two *Ciona* species, which have approximately the same sized genomes [12], [13] have different numbers of total markers, 175 and 356, but similar numbers of bands per individuals, 118±7 and 128±23, for *C. intestinalis* and *C. savignyi*, respectively. This difference in total bands is likely a reflection of the greater number of alleles in the *C. savignyi* population sampled versus the *C. intestinalis* population sampled. However, to make such comparisons between species that give differing average numbers of bands per individual the data are presented as the average number of bands per marker, which varied from 35 for *Styela plicata* and *B. schlosseri* (i.e., the average marker was found in 35 of 50 individuals sampled) to 18 for *C. savignyi*. A similar trend is seen if one examines the percentage of markers that are monomorphic (present in 50 out of 50 sampled individuals), shown in the third column of Table 2. These values range from 14.9% for *C. intestinalis* to 0.8% for *B. diegensis*.

**Table 2:** Calculated values from the similarity index for the ascidian species in the study.

A	Santa Barbara Ascidian Populations	Total Markers	Monomorphic Markers	Total Bands Scored	Average Bands/Marker	Average bands/individual±SD
	*Styela plicata*	233	29 (12.4%)	8152	35	163±10
	*Botryllus schlosseri*	328	24 (7.3%)	11406	35	228±15
	*Ciona intestinalis*	175	26 (14.9%)	5890	34	118±7
	*Botrylloides violaceus*	234	13 (5.6%)	7422	32	148±11
	*Aplidium californicum*	280	14 (5.0%)	8269	30	165±14
	*Styela clava*	261	24 (9.2%)	7230	28	145±11
	*Botrylloides diegensis*	389	3 (0.8%)	10631	27	213±27
	*Ascidia ceratodes*	353	17 (4.8%)	7430	21	149±12
	*Ascidia zara*	242	6 (2.5%)	5156	21	103±16
	*Ciona savignyi*	356	4 (1.1%)	6413	18	128±23

B	Ventura Ascidian Populations	Total Markers	Monomorphic Markers	Total Bands Scored	Average Bands/Marker	Average bands/individual±SD
	*Ciona intestinalis*	172	25 (14.5%)	5055	29	101±9
	*Ciona savignyi*	381	4 (1.1%)	7117	19	142±19

Part A of the table is for the ascidian species from SBYH. The species are sorted according to the values in the Average Bands/Marker row. Species which have higher numbers of average bands per marker have a higher degree of similarity and therefore lower levels of polymorphism. Part B contains the information from the Ventura *C. savignyi* and *C. intestinalis* populations.

**Figure 3:**
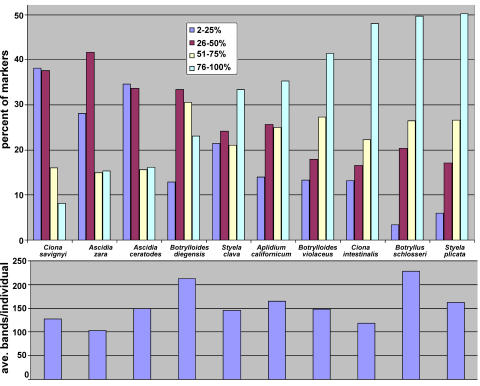
Graphical representation of ascidian genetic similarity. The top panel shows the percentage of the total markers for each population that are shared by indicated percent of the individuals sampled. The bottom panel shows the average number of bands per individual for the same data set and in the same species.

To present the results graphically, the markers for each species were put in four categories, those present in 25% or less of the 50 individuals sampled, those in 26–50% of individuals, those in 51–75% of individuals, and finally those in 76–100% of individuals. Those individuals in species with more markers present in a higher fraction of individuals sampled are thus more similar to each other. The results for all ten species are presented in Fig. 3 in rank order of those species with the greatest percentage of markers in the first two categories to those with the fewest. Thus for *C. savignyi* 75.8% of the markers were found in 50% or fewer of the individuals sampled, while only 23.3% of markers for *Styela plicata* fell in these categories. The order of species shown in Fig. 3 follows the same pattern as shown in Table 2A for the average bands per marker. The bottom panel of Fig. 3 shows the average number of bands per individual for the same data set. No correlation was observed between these two set of values, indicating that the relative degrees of similarity for the various species was not a function of the number of markers analyzed (i.e., simply having more markers did not make individuals within a species appear more or less similar by our measure). Thus we conclude that both the order of the species on Fig. 3 and in column 5 of Table 2A is a relative measure of similarity of individuals within a species, and is indicative of the number of alleles for the loci sampled, in other words, the relative polymorphism of the species (see Discussion).

The two *Ciona* species were nearly at the opposite extremes of polymorphism for the ten species examined, with *C. savignyi* being more polymorphic than *C. intestinalis*, in agreement with the genomic sequence data from single individuals from both species [13]. Comparisons of the two *Ciona* species in SBYH and VH show that the relative degrees of polymorphism between these two closely related species were similar at the two locations (Table 2B). The values for monomorphic alleles at these two locations, for example, are nearly identical.

### Population Structure Analysis

One potential factor influencing measured genetic variability of a population is the structure of that population. Structure is significant both within populations and between populations, and may have an impact on this study. The AFLP-SURV software package was used to calculate F_ST_ values between the *C. intestinalis* populations in SBYH and VH and the *C. savignyi* populations in those locations as well [21]. The resulting values (F_ST_ = 0.0806 and F_ST_ = 0.0097 respectively for the *C. intestinalis* and *C. savignyi* populations) show that there is not a high level of structure between these populations [22]. This observation reinforces the assumption that the populations of these ascidians in the SBYH are not atypical as compared to other harbors in the SCB.

**Figure 4:**
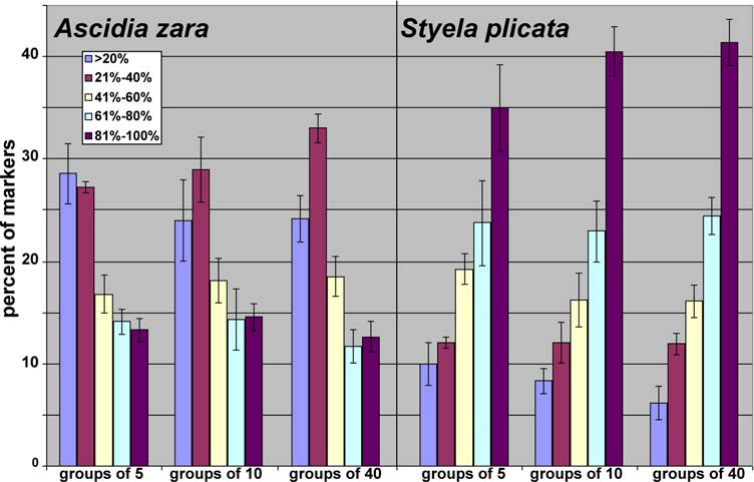
Representative resampling of similarity data for two ascidian species. For each species 5 replicates of data from 5, 10 and 40 randomly selected individuals were analyzed by the similarity matrix. The bars for the resampled data show the average of the 5 replicates (±standard deviation).

A second concern was that varying degrees of population structure between the ten species examined might distort the apparent differences in the similarity index (Fig. 3). We found that standard methods for addressing population structure [e.g., the *structure* software package [23]] did not perform well with samples from highly polymorphic species like ascidians (see Discussion). Instead to address the issues of sampling size and population structure, the data were resampled in groups as small as five individuals per group and compared to the full data set. Figure 4 shows representative examples of this analysis in which five groups of five, ten and forty individuals were examined. For the two species shown in Fig. 4, the relative degrees of similarity and the standard deviations among the five replicates in the resampled data were very similar between the differently sized pools (the other eight species had nearly identical results, not shown). This result is consistent with the ten species examined all having similarly low structure to their populations.

## Discussion

The motivation for the present study comes from the findings that the sequenced genomes of several marine invertebrate deuterostomes, in particular the ascidians *Ciona savignyi* and *Ciona intestinalis* and the urchin *Strongylocentrotus purpuratus*, reveal extremely high levels of heterozygosity [12], [13], [16], implying that these populations as a whole are highly polymorphic. Analysis of *C. savignyi* suggests that much of the heterozygosity can be accounted for by a large effective population size, rather than an elevated mutation rate [15]. While the sequenced genomes of the congeneric *C. savignyi* and *C. intestinalis* both show high heterozygosity, *C. savignyi* shows approximately 3.8-fold higher heterozygosity than *C. intestinalis*. In the present study we have used an AFLP-based method to further investigate the relative degrees of polymorphism among ten ascidian species found in southern California. AFLP has been used previously in similar studies, such as for *Morone* and *Thunnus* [24], which made use of a similarity index of interspecific populations to determine the level of polymorphism within these fish species. Also, Innan *et al.* [25] developed a method that used AFLP data to approximate the level of nucleotide diversity through the use of a similarity index.

In the present study, AFLP data was used to determine similarity between individuals of a species, which in turn allows us to compare polymorphism levels between species. The frequency of the presence allele for each marker of a species gives an indication as to the level of similarity within that species (Table 2). The higher the level of shared presence alleles within a population, the more similar the population is in regard to nucleotide diversity [25]. However, scoring for the presence or absence of a marker (e.g., Fig. 2) will give the amount of similarity within a population, which is not a direct measure of polymorphism, as it does not indicate the total number of alleles for any specific locus. Nevertheless, there is a strong link between similarity and polymorphism, with those populations which are more similar being less polymorphic. For the two *Ciona* species, the relative degrees of similarity agree well with their relative heterozygosity found in genomic sequences.

AFLP markers are dominant, so only a single allele must match between the two individuals in order for the marker to be present in both. Previous methods for analyzing AFLP data typically relied on the assumptions that AFLP produces genetic markers which are dominant and biallelic, having a single presence and null allele (reviewed in [26]). The frequency of these alleles could then be calculated by assuming Hardy-Weinberg equilibrium and using the formulas of p+q = 1 (with p being the frequency of the presence allele and q the frequency of the null) and p^2^+2pq+q^2^ = 1 (with p^2^ and q^2^ representing the homozygotes and 2pq being the heterozygotes). The absence of a band at a marker would be scored as an individual homozygous for the null allele. The frequency of the absence of a band at each marker could be used to then calculate the average frequencies of the presence and absence alleles for the population and a value of heterozygosity for the population could be calculated. One of the problems with this analysis method is the assumption that there are an equal number of null and presence alleles (one of each), and we know that this is very unlikely for a highly polymorphic species [27]. This makes calculations of p and q from this method unreliable in cases of high polymorphism. With the similarity matrix, by normalizing the number of markers into percent and calculating the level of shared markers as a percentage of the total markers, these values can be shown graphically and compared between species (Fig. 3). The data suggest a continuum of polymorphism levels among the ascidian species in the Santa Barbara Yacht Harbor, those species with a high percentage of shared bands being less polymorphic. By sampling *C. intestinalis* and *C. savignyi* in VH as well as SBYH, we have shown that the observed polymorphism levels of these species are very similar at these two locations. Through the measurement of the population structure between these two populations, it is found that there is not a large genetic distance between them.

Attempts to determine the population structure of the ascidian populations within SBYH using the *structure* 2.2 software were not successful (briefly, multiple values of “K” were found to have statistically identical probabilities). It is likely that the high level of null allele homoplasy found in the AFLP analysis of highly polymorphic species was the cause of this problem. However, the resampling data addresses the population structure question (although less quantitatively). If the populations were highly structured (i.e., composed of sub-groups that rarely cross and share alleles) it would be expected that resampled smaller groups would show considerable variation from the full data set, which was not observed for any species (Fig. 4). The resampling analysis suggests that the selected ascidian populations are not heterogeneous admixtures of multiple sub-populations, and that our analysis has captured the breadth of polymorphism present in SBYH. Furthermore, for the two species studied in greater detail, *C. savignyi* and *C. intestinalis*, very similar degrees of similarity were observed at two locations (nor do the two populations examined show much structure within the species). This is consistent with the *Ciona* genomic sequence data showing high heterozygosity within individuals. Thus, while we cannot rule out differing degrees of population structure may account for some of the observed differences in similarity between the species, it would not appear to be a major factor.

**Figure 5:**
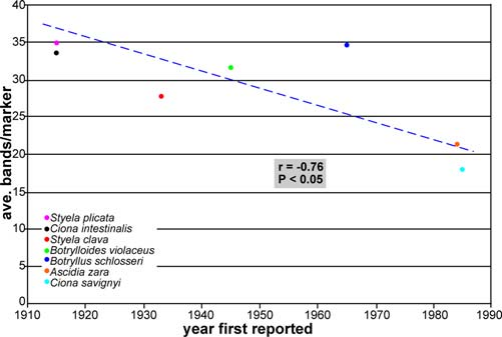
Plot shows the correlation between the date first reported and the calculated similarity level for the invasive ascidian species within the Southern California Bight. Surprisingly there was a negative correlation between date first recorded and estimated similarity level. It would be assumed that similarity would decrease as a population becomes more established, but this is not the case.

Having observed variable degrees of similarity between the ten ascidian species examined, we can begin to examine factors that might account for these differences. Typically, age, size, structure and mutation rate of the population are considered when trying to determine polymorphism level. However, lifestyle and fecundity could also play a role in determining population structure and polymorphism level, by affecting one of the previously mentioned factors. The majority of the ascidian species analyzed in this study are considered invasive to the Southern California Bight, many of which were first reported within the last 40 years [8], [9]. The invasive ascidians were likely present before the date they were first recorded, but either in numbers small enough that they were missed, or located in areas which were not a part of the surveys. At any rate, the ascidians recorded as invasive were not found in their present abundance in the relatively recent past. There is a moderately strong negative correlation (correlation coefficient = −0.7; P<0.05) among the invasive ascidian species between the time they were first recorded in the Southern Californian Bight and their current level of polymorphism (Fig. 5). Typically, polymorphism increases as a species or population ages and increases, but this may be a case where many individuals from highly polymorphic founder populations were introduced simultaneously and selective pressure has been slowly reducing the levels of polymorphism in order to maximize the abundance of beneficial alleles in these invaded populations.

Neither our current population surveys within SBYH (our unpublished data), nor previous surveys in southern California [8], [9], show any correlation of the calculated levels of polymorphism and population size. However, all surveys are limited in scope and could easily overlook large effective populations. Furthermore, historic population levels are likely to be highly variable, and our current sample will not reflect historical effective population size. It is believed that *C. intestinalis* are native to the North Atlantic while *C. savignyi* are native to Japan and the Western Pacific. Thus there may have been larger and more frequent reintroductions of *C. savignyi* to California from their founding population, but the current levels of polymorphism at the source populations of these species are not known. In addition no correlation was found between reproductive strategies and the degree of polymorphism. Those species which broadcast spawn their gametes have much higher levels of fecundity than do colonial ascidians, which brood their offspring until hatching, and consequently produce many fewer progeny. However, no correlation between fecundity and polymorphism level was apparent in our sample.

### Conclusion

In summary, neither population age or size, nor complexity of the AFLP fragment patterns (i.e., average number of bands per individual; Fig. 3, bottom), appear to account for the measured polymorphism level in these southern California ascidians. Complicated invasion patterns perhaps are obscuring these trends, or it may be that there are variable mutation rates among the different species which cause them to accumulate polymorphism at different rates. In any case, these invasive species are highly polymorphic and have become established relatively recently in southern California. This finding will hopefully be useful for others studying the population polymorphism levels of invasive species, or are trying to determine if species are invasive by looking at polymorphism. Analysis of the polymorphism levels of these species in their home range would be able to give a clearer picture as to the source of these differences in polymorphism levels.
